# TCR-engineered T cell therapy in solid tumors: State of the art and perspectives

**DOI:** 10.1126/sciadv.adf3700

**Published:** 2023-02-15

**Authors:** Estelle Baulu, Célia Gardet, Nicolas Chuvin, Stéphane Depil

**Affiliations:** ^1^ Centre de Recherche en Cancérologie de Lyon, Lyon, France.; ^2^ ErVaccine Technologies, Lyon, France.; ^3^ Centre Léon Bérard, Lyon, France.; ^4^ Université Claude Bernard Lyon 1, Lyon, France.

## Abstract

T cell engineering has changed the landscape of cancer immunotherapy. Chimeric antigen receptor T cells have demonstrated a remarkable efficacy in the treatment of B cell malignancies in hematology. However, their clinical impact on solid tumors has been modest so far. T cells expressing an engineered T cell receptor (TCR-T cells) represent a promising therapeutic alternative. The target repertoire is not limited to membrane proteins, and intrinsic features of TCRs such as high antigen sensitivity and near-to-physiological signaling may improve tumor cell detection and killing while improving T cell persistence. In this review, we present the clinical results obtained with TCR-T cells targeting different tumor antigen families. We detail the different methods that have been developed to identify and optimize a TCR candidate. We also discuss the challenges of TCR-T cell therapies, including toxicity assessment and resistance mechanisms. Last, we share some perspectives and highlight future directions in the field.

## Teaser

Engineering T cells with T cell receptors is a promising strategy to treat solid tumors.

## INTRODUCTION

Immunotherapy has revolutionized the therapeutic management of cancers in the past decade. Seven immune checkpoint inhibitors (ICIs) were Food and Drug Administration (FDA)–approved in more than 85 oncology indications in only 7 years (*1*). However, a large fraction of patients do not benefit from ICI due, in part, to the scarcity of tumor-specific effector T cells. This limitation could be overcome with adoptive cell transfer (ACT), which consists in the infusion of antigen-specific T cells in an amount much greater than what can be observed with endogenous response (*2*). Different ACT techniques are being developed, including tumor-infiltrating lymphocyte (TIL) therapy, T cell receptor–engineered T (TCR-T) cell therapy, and chimeric antigen receptor T (CAR-T) cell therapy. The ACT initially developed was based on the isolation of tumor-specific TILs for ex vivo expansion and reinfusion into the patient (*3*). While effective in certain cancer types such as melanoma, this approach was only feasible for resectable tumors from which enough T cells could be isolated and amplified (*4*). TCR-T and CAR-T cell therapies consist in genetically engineered T cells, modified to express a receptor directed against a tumor antigen. CAR-T cell therapies were a considerable breakthrough in hematological cancers, with six therapies now FDA-approved, targeting CD19 or B cell maturation antigen (*5*, *6*). However, the clinical efficacy of CAR-T cells in solid tumors has been much less rewarding, with multiple obstacles including the scarcity of available antigens, tumor heterogeneity, or tumor immunosuppression (*6*). Advanced solid tumors are also characterized by a desmoplastic stroma and an aberrant vascularization, resulting in hypoxia and altered nutrient availability (*7*, *8*). TCR-T cell therapy represents an alternative that offers several advantages. First, the repertoire of targetable antigens for TCR-T cell therapy is larger than for CAR-T cells. Indeed, because of the nature of the TCR, TCR-T cells can recognize epitopes derived from both membrane and intracellular proteins and presented by the major histocompatibility complex (MHC), while CAR-T cells are limited to targeting cell surface antigens. However, the antigen recognition for TCR-T cells is restricted to the human leucocyte antigen (HLA) allele presenting the epitope, thus restricting the number of patients who can benefit from a given TCR-T cell therapy. Second, the epitope density required to induce activation is lower for TCR-T cells than for classical CAR-T cells (1 to 50 versus 10^3^ epitopes per cell, respectively) (*9*). This increased sensitivity may improve tumor cell detection and killing. Last, the high avidity of TCR-T cells may also improve their efficacy, and the lower affinity of TCRs for their target compared to CARs may allow each TCR-T cell to “scan” and eliminate several antigen-presenting tumor cells.

To date, compelling clinical data were published for TCR-T cell therapies in solid cancers. In the following review, we perform a synthesis of the different types of tumor antigens now being targeted with TCR-T cell therapy in the clinic and new promising antigens developed at the preclinical stage. We review the different strategies to identify tumor-specific TCRs and optimize their expression and the efficacy of TCR-T cell therapy. We also discuss the challenges that need to be addressed to improve the safety and efficacy of this approach. Last, we give some perspectives for future research, presenting promising strategies to improve persistence of engineered T cells in vivo or to develop allogeneic approaches.

## THE REPERTOIRE OF TARGETABLE ANTIGENS IN CLINICAL TRIALS

**Fig. 1.**
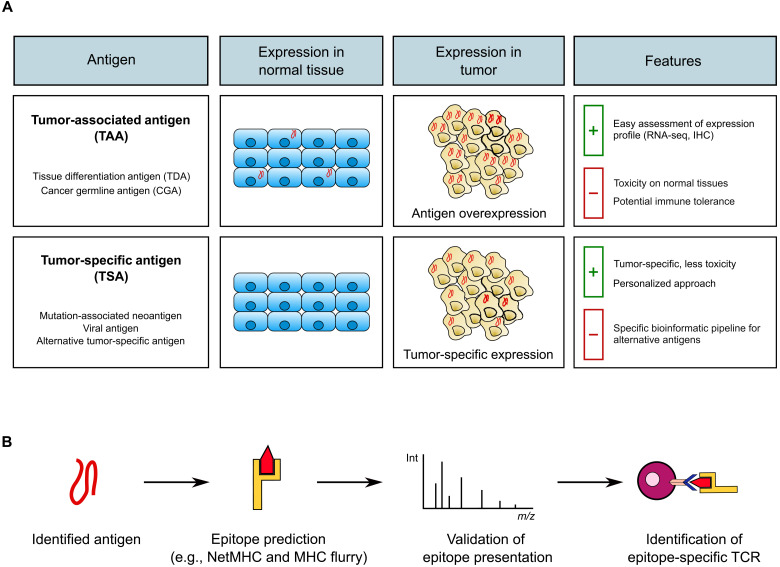
Choice of target antigen in TCR-T cell therapy. (**A**) Tumor-associated antigens (TAAs) are overexpressed in tumor versus normal tissues, whereas tumor-specific antigens (TSAs) are expressed exclusively in the tumor. Pros and cons of each antigen type are displayed in the last column. IHC, immunohistochemistry. (**B**) After identifying the antigen, epitopes need to be predicted on the basis of data-driven bioinformatic tools (examples: NetMHC and MHC Flurry) and their presence in tumor cells confirmed by peptidomics/immunopeptidomics. These steps are followed by the identification of epitope-specific TCRs. Int, intensity; *m*/*z*, mass/charge ratio.

Antigen selection is a key point in the development of safe and efficient TCR-T cell therapies. The ideal antigen would be expressed selectively and homogeneously in tumor cells and generate epitopes presented on MHC class I molecules on their surface. Two major classes of tumor antigens are now considered in clinical trials: tumor-associated antigens (TAAs) and tumor-specific antigens (TSAs) (Fig. 1) (*4*). Clinical trials for which results have been published are presented in Table 1.

**Table 1.** List of published TCR-T cell clinical trials. Symbols: (), response duration in months after treatment; +, ongoing. Abbreviations: TDAs, tissue differentiation antigens; CGAs, cancer germline antigens; CR, complete response; PR, partial response by RECIST criteria; CEA, carcinoembryonic antigen; gp100, glycoprotein 100; HPV, human papillomavirus; MAGE-A, melanoma-associated antigen; MART-1, melanoma antigen recognized by T cells 1; NY-ESO-1, New York esophageal squamous cell carcinoma-1; MPNST, malign peripheral nerve sheath tumors; NSCLC, non–small cell lung cancer; CRS, cytokine release syndrome; n.s., not specified; KO-KI, knockout–knock-in; N.A., not available.

Antigen type	Target antigen	Epitope	HLA	Cancer type	Vector	Number of cells	Number of patients	Clinical trial	Phase	Objective response rate (ORR) (%)	Clinical response (months)	Toxicities related to TCR-T cells (%)	Reference
**TDAs**	MART-1	AAGIGILTV	HLA-A*02:01	Melanoma	Retrovirus	0.5 to 34 × 10^9^	17	n.s.	n.s.	2 (12%)	2 PR (20, 21)	None	Morgan *et al.* (*10*)
	MART-1	AAGIGILTV	HLA-A*02:01	Melanoma	Retrovirus	0.15 to 10 × 10^10^	20	NCT00509288	II	6 (30%)	6 PR (3, 4, 9, 17+, 17+)	Skin rash: 14 (70%)	Johnson *et al.* (*11*)
												Uveitis: 11 (55%)	
												Hearing loss: 10 (50%)	
	MART-1	EAAGIGILTV	HLA-A*02:01	Melanoma	Retrovirus	0.6 to 4.8 × 10^9^	13	NCT00910650	II	0		Skin rash: 3 (23%)	Chodon *et al.* (*12*)
												CRS: 2 (15%)	
	MART-1	EAAGIGILTV	HLA-A*02:01	Melanoma	Retrovirus	0.05 to 4.56 × 10^9^	12	NCT02654821	I/IIa	2 (16.7%)	2 PR (4.1, 7.1)	CRS/sepsis: 1 (8%)	Rohaan *et al.* (*13*)
												Dermatitis: 10 (83%)	
												Uveitis: 2 (17%)	
												Hearing loss: 4 (33%)	
	gp100	KTWGQYWQV	HLA-A*02:01	Melanoma	Retrovirus	0.18 to 11 × 10^10^	16	NCT00509496	II	3 (16%)	1 CR (14+)	Skin rash: 15 (94%)	Johnson *et al.* (*11*)
												Uveitis: 4 (25%)	
											2 PR (4, 3)	Hearing loss: 5 (31%)	
	CEA	IMIGVLVGV	HLA-A*02:01	Colorectal cancer	Retrovirus	2 to 4 × 10^8^	3	NCT00923806	I	1 (33%)	1 PR (*6*)	Severe transient colitis: 3 (100%)	Parkhurst *et al.* (*14*)
**CGAs**	MAGE-A3	KVAELVHFL	HLA-A*02:01	Melanoma	Retrovirus	2.8 to 7.9 × 10^10^	9 (7 + 1 + 1)	NCT01273181	I/II	5 (56%)	1 CR (15+)	Severe neurologic toxicity: 3 (33%) including 2 deaths	Morgan *et al.* (*16*)
				Synovial sarcoma							4 PR (4, 4, 5, 12+)		
				Esophageal cancer									
	MAGE-A3	EVDPIGHLY	HLA-A*01	Melanoma	Lentivirus	2.4 to 5.3 × 10^9^	2 (1 + 1)	NCT01350401	I	0		Severe cardiac toxicity and death: 2 (100%)	Linette *et al.* (*17*)
				Myeloma				NCT01352286					
	MAGE-A3		HLA-DPB1*0401	Metastatic solid tumors	Retrovirus	0.01 to 123 × 10^9^	17	NCT02111850	I	4 (23.5%)	1 CR (29+) 3 PR (4, 4, 18+)	Liver toxicity: 2 (12%)	Lu *et al.* (*18*)

	MAGE-A4	NYKRCFPVI	HLA-A*24:02	Esophageal cancer	Retrovirus	0.2 to 5 × 10^9^	10	UMIN000002395	I	0	none	Kageyama *et al.* (*19*)	
	MAGE-A4	GVYDGREHTV	HLA-A*02	Advanced solid tumors	Lentivirus	0.12 to 10 × 10^9^	38	NCT03132922	I	9 (23.7%)	9 PR	CRS: 9 (50%)	Hong *et al.* (*20*)
	MAGE-A10	GLYDGMEHL	HLA-A*02:01 or HLA-A*02:06	NSCLC	Lentivirus	0.1 to 6.77.10^9^	11	NCT02592577	I	1 (9%)	1 PR (1)	CRS: 3 (27%)	Blumenschein *et al.* (*21*)
												Neurotoxicity: 1 (9%)	
	NY-ESO-1	SLLMWITQC	HLA-A*02:01	Melanoma	Retrovirus	0.16 to 13 × 10^10^	17 (11 + 6)	NCT00670748	I	5 (45%) 4 (67%)	2 CR (20+, 22+)	None	Robbins *et al.* (*22*)
				Synovial sarcoma							3 PR (3, 8, 9+)		
											4 PR (5, 8, 10, 18)		
	NY-ESO-1	SLLMWITQC	HLA-A*02:01	Melanoma	Retrovirus	0.9 to 13 × 10^10^	38 (20 + 16)	NCT00670748	II	11 (55%) 11 (61%)	4 CR (24, 40+, 54+, 58+)	None	Robbins *et al.* (*23*)
											7 PR (3, 3, 5, 6+, 8, 10, 28)		
				Synovial sarcoma							1 CR (6)		
											10 PR (3, 3, 4, 5, 7, 8, 10, 11, 18, 47+)		
	NY-ESO-1	SLLMWITQC	HLA-A*02:01	Melanoma	Retrovirus	1 × 10^9^	10	NCT02070406	I	2 (20%)	2 PR	CRS: 1 (10%)	Nowicki *et al.* (*24*)
				Synovial sarcoma									
				Liposarcoma				NCT01697527					
				Osteosarcoma									
				MPNST									
	NY-ESO-1	SLLMWITQC	HLA-A*02:01 HLA-A*02:06	Synovial sarcoma	Lentivirus	0.4 to 14.4 × 10^9^	12	NCT01343043	I/II	6 (50%)	1 CR (8)	CRS: 5 (42%)	D’Angelo *et al.* (*25*)
											5 PR (4, 7, 8, 18)		
		NY-ESO-1SLLMWITQC	HLA-A*02:01 HLA-A*02:06	Synovial sarcoma	Lentivirus	2.67 × 10^9^	30	NCT01343043	I/II	9 (30%)	9 PR (2-13)	n.s.	Ramachandran *et al.* (*26*)
		NY-ESO-1(CRISPR -Cas9)SLLMWITQC	HLA-A*02:01	Liposarcoma Myeloma	Lentivirus	0.6 to 7.1 × 10^8^	3	NCT03399448	I	0		None	Stadtmauer *et al.* (*27*)

**Viral antigens**	HPV16-E6	TIHDIILECV	HLA-A*02:01	HPV16-positive epithelial cancer	Retrovirus	0.1 to 13.4 × 10^10^	12	NCT02280811	I/II	2 (17%)	2 PR (3, 6)	None	Doran *et al.* (*38*)
	HPV16-E7	YMLDLQPET	HLA-A*02:01	HPV16-positive epithelial cancer	Retrovirus	0.1 to 12.10^10^	12	NCT02858310	I	6 (50%)	6 PR (3, 4, 4, 8, 8, 9)	None	Nagarsheth *et al.* (*39*)
	HBV	N.A.	HLA-A*02 or HLA-Cw0801	HBV-HCC	Electroporation	1 × 10^4^/kg to 5 × 10^6^/kg	8	NCT03899415	I	1	1 PR (27.7)	Liver toxicity: 1 (12%)	Meng *et al.* (*40*)
	MCPyV	KLLEIAPNC	HLA-A*02:01	Merkel cell carcinoma	Lentivirus	1 to 9 × 10^8^	5	NCT03747484	I	1 (25%)	n.s.	None	Veatch *et al.* (*41*)
**Neo-antigens**	TP53	HMTEVVRHC	HLA-A*02:01	Metastatic breast cancer	Retrovirus	5.3 × 10^10^	1	NCT03412877	I	1 (100%)	1 PR (6)	CRS	Kim *et al.* (*34*)
	KRAS G12D	GADGVGKSA	HLA-C*08:02	Metastatic pancreatic cancer	Retrovirus	1.6.10^10^	1	IND 27501	I	1 (100%)	1 PR (6+)	None	Leidner *et al.* (*35*)
		GADGVGKSAL											
	Mutation-associated neoantigens (CRISPR-Cas9 KO-KI)		Multiple HLA class I	Metastatic solid tumors	Electroporation	0.13 to 4.10^9^	16	NCT03970382	I	0		CRS: 1 (6%)	Foy *et al.* (*37*)
												Neurotoxicity: 1 (6%)	

### Tumor-associated antigens

TAAs are antigens overexpressed in cancers but with a limited expression in normal tissues. Their expression can be restricted to tissues of tumor origin [tissue differentiation antigens (TDAs)] or to germline tissues [cancer germline antigens (CGAs)] (*4*). TAAs are attractive therapeutic targets because they are often shared between patients. However, because of their expression in normal tissues, although at a low level, they may be associated with some on-target off-tumor toxicity. In addition, high-affinity–specific T cells may be eliminated during thymic negative selection, making the identification of potent TCRs more difficult.

#### Tissue differentiation antigens

Clinical trials targeting TDAs, such as melanoma antigen recognized by T cells 1 (MART-1) (*10*–*13*), glycoprotein 100 (gp100) (*11*), or carcinoembryonic antigen (CEA) (*14*), showed some clinical responses, but several toxicities were described because of their low expression in normal tissues. In the first clinical trial evaluating MART-1–specific TCR-T cells in patients with melanoma, the objective response rate (ORR) did not exceed 12% (2 of 17) (*10*). To improve the clinical response, an affinity-enhanced TCR-recognizing MART-1 was tested in two other clinical trials. Although clinical response was slightly improved in one of the trials, with ORR of 30% (6 of 20) and 0% (0 of 13), respectively, several serious cutaneous, ocular, and auditive toxicities were described because of low expression of MART-1 in normal melanocytes (*11*, *12*). More recently, a clinical trial using a different TCR for MART-1 had to be prematurely terminated because of severe toxicities as described before and the death of one patient (*13*). Similar results were observed with TCR-T cells targeting gp100. The ORR was 16% in patients with melanoma (3 of 16), but, as previously observed, many cutaneous, ocular, or auditive adverse events were reported (*11*). It should be mentioned in this context that tebentafusp, a soluble affinity-enhanced TCR specific for gp100 fused to an anti-CD3 single-chain variable fragment, has been approved for the treatment of HLA-A*02:01–positive adult patients with unresectable metastatic uveal melanoma, on the basis of the results of a phase 3 randomized trial showing a substantial overall survival benefit. Toxicity was mostly mild to moderate, with most treatment-related adverse events classified as either skin-related (because of gp100-positive melanocytes) or cytokine-mediated (because of T cell activation) (*15*). In clinical trials evaluating TCR-T cells targeting CEA, one of the three patients with colorectal cancer experienced a partial response, but side effects such as severe inflammatory colitis were described in all patients (*14*). Other targets, such as mesothelin in pancreatic cancer, are now being tested in the clinic (NCT04809766).

#### Cancer germline antigens

Most of the TCR-T cell clinical trials targeting CGAs, also called “cancer testis antigens,” focus on members of the melanoma-associated antigen (MAGE-A) protein family (*16*–*21*) and New York esophageal squamous cell carcinoma-1 (NY-ESO-1) (*22*–*27*). The first two clinical trials targeting two different MHC class I–restricted epitopes derived from MAGE-A3 led to severe and lethal toxic effects caused by cross-reactivity (recognition of an unrelated epitope). In the first one, an objective response was observed in 56% of patients (five of nine), but MAGE-A3 TCR-T cells cross-reacted with MAGE-A12, a protein expressed in the brain, causing severe neurotoxicities and two deaths (*16*). The second trial was prematurely stopped because of cardiac toxicities and the death of the two treated patients. It was retrospectively determined that MAGE-A3–specific TCR-T cells also recognized an epitope derived from TITIN, a protein expressed in cardiomyocytes (*17*, *28*). More recently, better clinical results were obtained with MHC class II–restricted TCR targeting MAGE-A3, with 25.3% ORR (4 of 17) and no major toxicities (*18*). MAGE-A4 was also targeted using TCR-T cells. No clinical response was observed in a first trial performed in patients with esophageal cancer (*19*). However, very encouraging results were recently reported with affinity-enhanced TCR-T cells targeting a different MAGE-A4 epitope (afamitestine autoleucel), especially in sarcoma. At the time of presentation of the phase 2 trial SPEARHEAD-1, the evaluable population included 33 patients with synovial sarcoma and 4 patients with myxoid/round cell liposarcoma. The ORR was 39.4%, with a disease control rate of 84.8%. Two complete responses were observed in patients with synovial sarcoma. Toxicity was manageable with cytokine release syndrome (CRS) in 22 patients (59%) (*20*, *29*). Preliminary clinical results of a strategy targeting MAGE-A10 in patients with non–small cell lung cancer were less encouraging because only 1 of the 11 patients experienced a transient partial response (ORR, 9%), and adverse events including CRS and neurotoxicities were described (*21*).

TCR-T cells targeting NY-ESO-1 showed promising results in clinical trials, especially in melanoma and synovial sarcoma. Among 107 patients treated in five clinical trials, the average response rate was 47% (ORR between 20 and 67%) with 8 complete responses and 40 partial responses without major toxicities (*23*–*26*). In a recent clinical trial using NY-ESO-1 TCR-T cells, endogenous TCR and programmed cell death 1 (PD-1) were knocked out using the CRISPR-Cas9 genome editing tool. Although no clinical response was observed among the three treated patients, in vivo persistence of the CRISPR-engineered T cells was increased (36 weeks versus 1 week) compared to other trials studying NY-ESO-1 (*27*). While showing promising results, the prevalence of NY-ESO-1 expression is still limited in metastatic cancers, and its tumor expression is often heterogeneous (*30*). Other CGAs are being tested in the clinic, such as KK-LC-1 (NCT03778814 and NCT05035407) or PRAME (NCT03686124 and NCT02743611), with no clinical results yet published.

### Tumor-specific antigens

TSA, also called neoantigens, are proteins exclusively expressed by tumor cells because they are linked to the tumorigenesis process (mutations and viral induction). Targeting these neoantigens with immunotherapy presents a very limited risk of toxicity because they are not expressed by normal tissues. Moreover, high-avidity T cells specific for these neoantigens are not eliminated during the negative thymic selection and can be isolated from patient tumor or healthy donor peripheral blood (*31*).

#### Mutation-associated neoantigens

These neoantigens result from nonsynonymous mutations linked to cancer-initiating genetic events or to global genetic instability (*32*). “Public” neoepitopes refer to epitopes derived from frequently mutated driver genes, such as *TP53*, *KRAS*, or *PIK3CA* (*31*), that will be shared between different patients with one specific HLA allele. TCR-T cells targeting these public neoantigens are now being tested in clinical trials. In a recent study, a screening was performed to identify neoepitopes derived from shared *TP53* mutations and corresponding specific TCRs. One patient with breast cancer was treated with TCR-T cells targeting a p53^R175H^ neoepitope and experienced a partial response with limited toxicity. However, the patient progressed after 6 months because of loss of class I MHC expression (*33*, *34*). For *KRAS*, a single-patient investigational new drug application was performed to evaluate the safety and tolerability of KRAS^G12D^-specific TCR-T cells in a patient with pancreatic cancer. After 6 months, tumor regression was still ongoing with no toxicity described, and functional TCR-engineered T cells were persisting in the circulation (*35*). TCR-T cells targeting the KRAS^G12V^ mutant (Mut) are also being tested in the clinic (NCT03190941). Last, screening methods were used to identify TCRs that recognize a Mut PIK3CA public neoantigen shared among HLA-A*03:01 patients. Engineered Mut PIK3CA–specific TCR-T cells showed an antitumor response against established tumors in vivo in mice bearing PIK3CA-Mut tumor but not wild-type PIK3CA tumors (*36*). Together, these results demonstrate the clinical interest of targeting neoantigens from mutated cancer drivers with TCR-T cells therapy. However, identifying epitope containing the public mutation with the HLA restriction limits the list of potential target epitopes. This issue may be partially addressed by targeting passenger mutations that occur in genes that do not intervene in the carcinogenic process due to cancer genetic instability. The development of a personalized TCR-T cell therapy is challenging and even more complex than for cancer vaccines. A pioneering clinical study has recently been published, demonstrating the feasibility of using CRISPR gene editing to create personalized TCR-T cells (*37*). For each of 16 trial participants, neoantigen-specific TCR were isolated, cloned, and validated from each individual’s blood. The two endogenous TCR genes from the patients’ own T cells were deleted, and the sequences encoding the selected neoantigen-specific TCR were simultaneously inserted. Patients received up to three different TCR-T cells in a cell dose escalation. No major safety concerns were observed. The authors showed that the TCR-T cells migrated to the tumors. Five patients experienced stable disease, and the other 11 had disease progression as best response on therapy. Despite moderate clinical activity, this study paves the way for the development of optimized personalized TCR-T cell therapies. Combination with anti–PD-1 antibody is now being tested in the clinic (NCT03970382 and NCT04520711).

#### Viral antigens

Some cancers can be induced by viral infections, such as human papilloma virus (HPV)–, hepatitis B virus (HBV)–, Merkel cell polyomavirus (MCPyV)–, or Epstein-Barr virus (EBV)–associated cancers. E6 and E7 viral antigens expressed in HPV-associated cancers have been targeted in clinical trials. In the first trial using HPV16-E6–specific TCR-T cells, clinical responses were reported in 17% of patients (2 of 12) without apparent toxicities (*38*). In the second trial using HPV16-E7 TCR-T cells, partial responses were observed in 50% of patients (6 of 12) up to 9 months after treatment with no notable toxicities. Nevertheless, tumor escape due to decrease of antigen presentation was described in several patients (*39*). Recently, HBV-specific TCR-T cells targeting HBV-associated hepatocellular carcinoma were tested in the clinic. One of the eight patients (ORR, 12.5%) experienced a partial response lasting 27.7 months, with minor toxicities described (*40*). Preliminary results of MCPyV-specific TCR-T cells for the treatment of PD-1 inhibitor-refractory metastatic Merkel cell carcinoma showed a 25% ORR, with one of the five patients presenting a mixed response. However, other effective therapies have been administered at the same time, making it difficult to interpret the results (*41*). TCR-T cells targeting other viral proteins are now tested in the clinic, such as latent membrane proteins (LMP1 and LMP2) in EBV-related nasopharyngeal carcinoma (NCT03925896, NCT04509726, and NCT03648697). Even if the applications are limited to some cancers, viral antigens benefit from the inherent tumor specificity with no toxicity toward normal tissues described in current clinical trials. In addition, they can be shared between patients with the same viral-induced cancer and HLA type (*9*).

#### Alternative TSAs

Alternative processes can generate TSAs derived from the noncoding genome; from alternate open reading frames; or from aberrant transcription, translation, or posttranslational modifications (*31*, *42*–*44*), and are referred to as “alternative tumor-specific antigens” (ATSAs) (*42*). One example of ATSA is represented by mutational frameshift neoantigens, which derive from peptides generated by frameshift insertions/deletions (INDELs). Roudko *et al.* (*45*) identified that patients with microsatellite instability–high tumors shared tumor-specific frameshift mutations resulting from INDELs within microsatellite sequences. Neoepitopes derived from these frameshift mutations are shared between patients and present a strong immunogenicity in vitro. Tumor antigens can also arise from abnormal mRNA splicing, such as intron retention or exon-exon junctions (*44*). Aberrant translation can also generate ATSA. As an example, Charpentier *et al.* (*46*) identified specific melanoma neoantigens, MELOE-1 and MELOE-2, derived from internal ribosome entry site–dependent translation of *meloe* long noncoding RNA. These neoantigens, for which specific TILs from patients with melanoma have been identified, are highly immunogenic in vitro. Development of identification techniques for alternative open reading frame or translation of “noncoding” sequences enabled the discovery of so-called “cryptic antigens” (*43*). Using mass spectrometry (MS) analysis of MHC class I immunopeptidome, Laumont *et al.* (*47*) estimated that cryptic antigens represented ~10% of MHC class I epitopes in leukemia patients. More recently, Ouspenskaia *et al.* (*48*) performed the ribosome profiling of 29 primary healthy and cancer samples and cell lines to create a database for MS identification of cryptic antigens. Results showed that new or unannotated open reading frames (nuORFs) contribute to 1.5 to 2.2% of MHC class I immunopeptidome in 10 cancer samples, with 50% of nuORFs detected in more than 1 sample. Retroelements also constitute a source of ATSA (*42*). Human endogenous retroviruses (HERVs) result from ancient retroviral infections and represent 8% of the human genome. Usually epigenetically silenced in normal tissues, HERVs can be reactivated in tumors due to DNA demethylation. Some HERVs are exclusively expressed in tumors, such as HERV-E in renal cell carcinoma (RCC). After showing preclinical antitumoral activity, HERV-E TCR-T cells are now being tested in the clinic in patients with RCC (*49*). Although no clinical data are available for these unconventional antigens, these findings increase the repertoire of potential targetable tumor antigens for TCR-T cell therapy.

## TCR IDENTIFICATION AND OPTIMIZATION

**Fig. 2.**
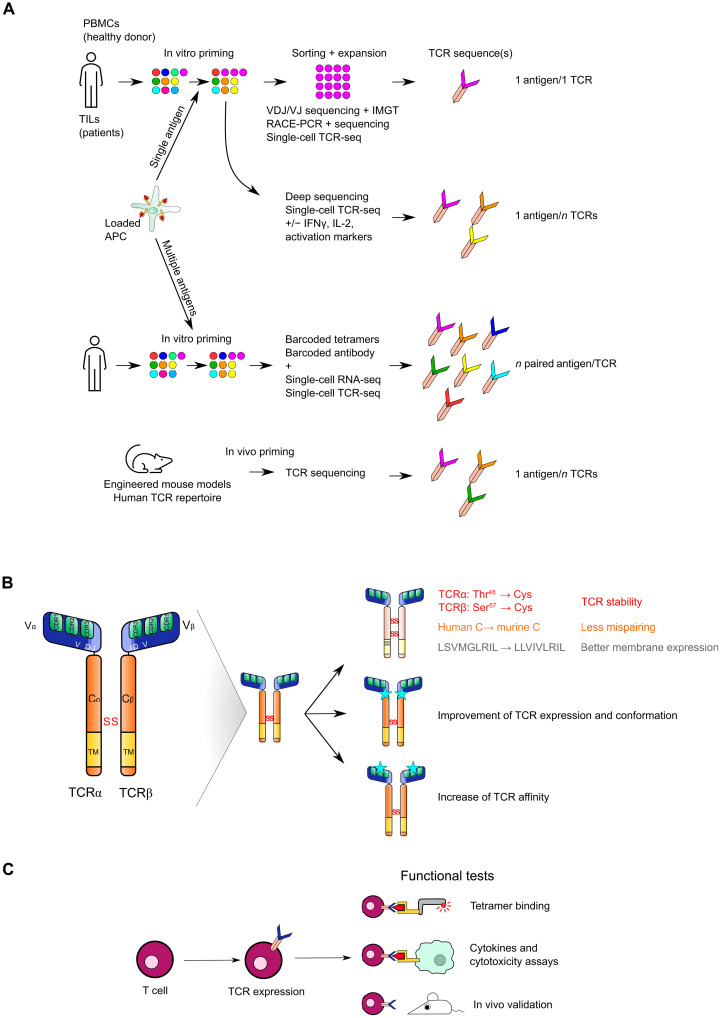
TCR identification, optimization, and validation. (**A**) T cells from healthy donor’s peripheral blood mononuclear cells (PBMCs) or patient’s TILs can be primed by antigen-presenting cells (APCs) loaded with one or several epitopes or antigens to stimulate the activation and expansion of specific T cells. TCRs from these specific T cells can be identified by sequencing T cell bulk or single cells. RACE-PCR, rapid amplification of cDNA ends–polymerase chain reaction; IFN-γ, interferon-γ. (**B**) TCR optimization can be performed by modifying the constant regions of both TCRα and TCRβ chains to prevent mispairing and increase TCR expression and stability. More recently, mutations in the variable regions but outside CDR regions have been shown to improve TCR expression and stability. TCR affinity maturation aims at increasing TCR affinity. (**C**) Once the TCR has been identified, in vitro and in vivo functional assays validate the specificity and the efficacy of the engineered T cells.

Identification of an epitope-specific TCR is complex given the extent of the TCR repertoire and the characteristics of the interaction between the TCR and the peptide-MHC (pMHC) complex. Indeed, it is estimated that one TCR can recognize up to 10^6^ different epitopes (*50*), and one epitope can be recognized by several TCRs (*51*). Identification techniques of pMHC-specific TCRs are based on in vitro induction of a T cell response in a specific HLA context. T cells can be isolated directly from a tumor or from patient’s or healthy donors’ blood. The first step typically consists in performing antigen stimulation of T cells (T cell priming) to enrich the population with antigen-specific T cells after clonal expansion (Fig. 2A). Then, epitope-specific T cells can be sorted and amplified in vitro. TCR sequences can be determined by TCRα and TCRβ sequencing of isolated T cells after rapid amplification of cDNA ends–polymerase chain reaction. (*52*–*54*). More recently, some groups combined VDJ sequencing and reference sequences from the ImMunoGeneTics database to identify functional TCRs (*55*, *56*). The development of single-cell RNA sequencing (scRNA-seq) focused on TCR sequences (scTCR-seq) has also facilitated the identification of TCRs from primed T cells. ScTCR-seq was combined with deep sequencing to identify TCR sequences of T cells expressing high levels of activation markers after antigenic stimulation, therefore selecting highly functional TCRs early in the process (*57*, *58*). Combination of barcoded tetramers, barcoded antibodies, and scRNA-seq allowed the identification of functional TCRs with peptide-specific activation signatures (*59*). The evolution of these techniques opens the path to simultaneously identify TCRs specific for different epitopes through the transduction of tandem minigenes in antigen-presenting cells (APCs) or by pulsing APCs with peptide libraries (*58*, *60*). Moreover, the recent advances in in vivo mouse models with a humanized T cell repertoire created new opportunities to identify human epitope-specific TCRs after in vivo priming/vaccination (*61*, *62*). One advantage of these models is the increased likelihood of identifying high-affinity TCRs in animals where the absence of antigen expression prevented negative thymic selection (*63*). However, TCR identification from human T cells may limit the risk of selecting self-reactive T cells.

Once the TCR sequence has been identified, optimization is often performed to improve TCR expression and/or affinity (Fig. 2B). For instance, some TAA-specific TCRs may have a low affinity due to elimination of high-affinity T cells specific for these self-antigens during thymic selection (*64*). Because T cell cytotoxic activity depends on TCR affinity (*65*), different methods were developed to improve TCR affinity, such as random mutation of the pMHC recognition site, phage display, or identification of high-affinity TCRs from immunization of humanized mice with the target epitope. Nonetheless, increasing TCR affinity is a double-edged sword (*64*). The capacity of T cells to eliminate a tumor cell presenting the epitope of interest depends on the cytotoxic response at the immune synapse and on TCR-pMHC interaction lability that allows T cells to eliminate several targets successively. A TCR with too strong affinity may lead to the early exhaustion of T cells following target antigen recognition, limiting successive tumor cell recognition. In addition, modifying TCR sequence may also bypass the negative selection that occurs in the thymus and thus create unanticipated cross-reactivity with self-antigens, as detailed below (*11*, *12*, *14*, *16*, *17*).

High TCR expression and proper assembly is also key in the generation of TCR-T cells. Recent work highlighted the importance of the global TCR conformation and especially the interactions between the variable and constant regions in proper TCR expression. Thomas *et al.* (*66*) demonstrated that the high variability of TCR expression and assembly, despite equivalent initial characterization, relies on specific residues at the structural interface between the variable and the constant regions. Replacing suboptimal residues at specific positions by optimal amino acids resulted in homogenization of TCR expression levels. The impact on TCR specificity was not addressed. Other TCR sequence engineering can improve expression and stability of the exogenous/transgenic TCR when expressed in T cells. Highlighted by Rosenberg’s team, these modifications increase the functionality of the engineered T cells (Fig. 2B). Substitution of human constant regions of both TCRα and TCRβ chains by the corresponding murine constant regions is essential to avoid mispairing and does not lead to toxicities in the clinic (*67*). Indeed, without these substitutions, exogenous TCRα and TCRβ chains can respectively pair with endogenous TCRβ and TCRα chains, leading to the creation of new and potentially cross-reactive TCRs. The addition of a second disulfide bond in the murine constant region increases exogenous TCR stability at the membrane (*68*), while addition of a hydrophobic sequence within the α chain transmembrane region facilitates membrane expression (*69*).

Recently, new approaches with CRISPR-Cas9 technology were used to replace the endogenous TCR with the transgenic TCR. Genetic knockout (KO) of both endogenous α and β chains circumvents the mispairing issues and prevents competitive binding to the CD3 complex with the endogenous TCR (*70*, *71*). However, although no serious adverse events have been described in clinical trials using genome editing so far, recent findings highlight the need to carefully assess genome integrity when using the CRISPR-Cas9 technology (*72*). Endogenous TCR KO can be combined with different gene delivery methods. Retroviral or lentiviral vectors are commonly used. These viral methods lead to the random integration of *n* copies of the TCR transgene in the host genome. Using homology DNA repair instead of lentiviral transduction for the knock-in (KI) of the transgenic TCR at the *T cell receptor* α *constant* (*TRAC*) locus limits the number of transgenic copies to one, resulting in a more homogenous product with decreased risk of oncogenic genetic events (*73*) and a physiological expression of transgenic TCR (*74*). However, the insertion rate is very low with this approach (5 to 10% of T cells). Recently, endogenous TCR KO by CRISPR-Cas9 technology was combined with adeno-associated virus (AAV) transduction to perform a KI of the TCR transgene at the TRAC locus. This enabled to substantially increase the insertion rate (60 to 70% of CD3^+^ T cells) while maintaining a physiological expression of the exogenous TCR. These KI TCR-T cells showed increased functional avidity and reduced cross-reactivity in vivo (*75*). These results are similar to those obtained with CAR-T cells, where the insertion of the CAR transgene at the TRAC locus by AAV vectors enhanced CAR-T cell potency. It also induced an effective recycling of the receptor, contributing to delayed exhaustion of the engineered T cells (*76*, *77*). Nonviral gene delivery is developing rapidly with the promises of faster and less expensive development, the possibility to modify several endogenous genes at one time and an improved safety. In the recent publication of Foy *et al.* (*37*) reporting the engineering of personalized TCR-T cells, the two TCRα and TCRβ chains were removed using CRISPR-Cas9 technology, and the sequences of the neoantigen-specific TCR were simultaneously inserted using a nonviral approach, with a KI efficacy reaching 23% (range, 11.4 to 46.8%) with the optimized process developed during the course of the study.

The selected T cell engineering strategy needs to be validated in functional assays to evaluate TCR-T cell efficiency and safety in vitro and in vivo (Fig. 2C) (*64*, *78*). Multimer binding assays validate proper TCR assembly and conformation. Assessment of cytotoxicity and cytokine release in coculture experiments with target cells and tumor cells allows assessing TCR-T cell efficacy, specificity, and avidity. Administration of TCR-T cells in engineered mouse models is a prerequisite to validate TCR-T cell viability, functionality, and persistence in vivo (tumor cell infiltration and formation of a memory compartment). A detailed review of all the models and options available to evaluate efficacy and safety has been made in two publications of the T^2^EVOLVE consortium (*79*, *80*).

## CHALLENGES OF TCR-T CELL THERAPIES

### Toxicity prediction

**Fig. 3.**
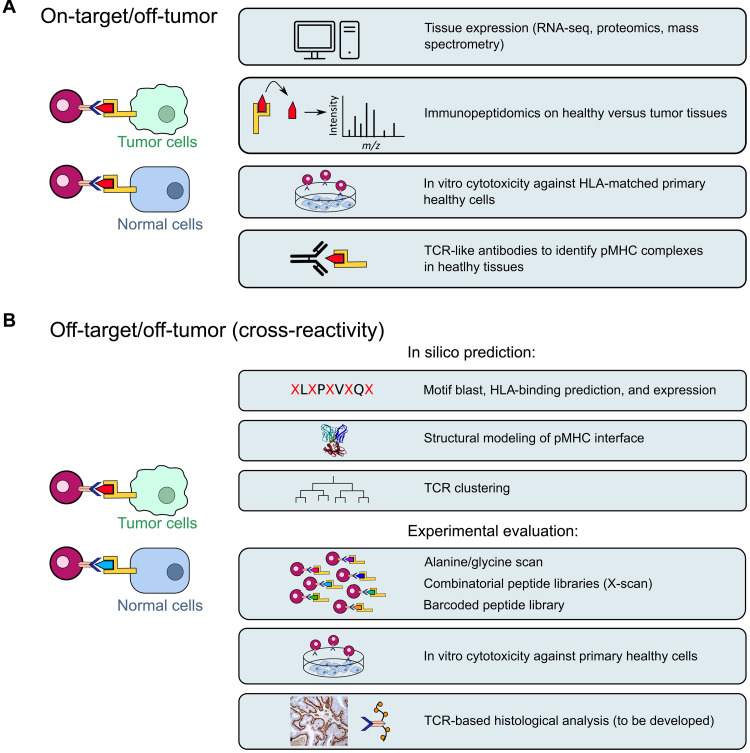
Methods to assess the potential toxicities of TCR-T cell therapies. (**A**) Methods to anticipate on-target off-tumor toxicities (presence of the targeted epitope in normal tissues). (**B**) Methods to anticipate off-target off-tumor toxicities (presence of a cross-reactive epitope in normal tissues).

Several toxicities associated with TCR-T cells have been described in the clinic. On-target off-tumor toxicities are linked to target antigen expression in normal tissues and are mostly associated with TAAs. In clinical trials targeting MART-1 and gp100 with TCR-T cell therapy, ocular, cutaneous, and auditive toxicities were due to TAA expression in melanocytes (*11*, *12*). Another study described severe acute colitis in patients treated with TCR-T cells targeting CEA due to its expression on normal intestinal cells (*14*). Evaluation of TCR-T cell–related on-target off-tumor toxicity includes bioinformatic analysis of transcriptomic and proteomic databases, immunopeptidomics, and in vitro or ex vivo assays to assess the capacity of TCR-T cells to recognize normal cells or tissues (Fig. 3A). The recent development of CAR-T cells targeting pMHC complexes (*81*) may also allow to develop pMHC-specific antibodies for histological assessment of epitope presentation in tumor versus normal tissues.

Off-target off-tumor toxicities or cross-reactivity are linked to the TCR recognition of a different antigen than the targeted antigen on normal cells. Cross-reactivities have been reported in clinical trials using affinity-enhanced TCR-T cells, as previously mentioned. Indeed, TCR-T cells targeting MAGE-A3 also recognized an epitope derived from MAGE-A12 or TITIN proteins, leading to the death of four patients (*16*, *17*, *28*). Therefore, it is essential to include a strategy evaluating TCR cross-reactivity at the preclinical level, especially when TCR sequences have been modified to enhance affinity (Fig. 3B). Initial assessment of cross-reactive epitopes can consist in the consecutive replacement of each peptide residue by an alanine residue (alanine scan) to identify residues essential for TCR-pMHC interactions (*82*). These essential residues can also be defined by bioinformatics as any combination of five to six amino acids of the 9- or 10-mer epitope (*83*). Peptide motifs composed of essential amino acids are then blasted against the human proteome to identify other proteins containing similar motifs that can serve as epitopes binding HLA molecules. This method succeeded in identifying retrospectively the cross-reactivity between MAGE-A3 and TITIN epitopes responsible for fatal cardiac toxicities. However, it does not take into account cross-reactivities that result from the replacement of residues at the same position by amino acids with similar physicochemical properties (*84*). In this context, other teams developed a mutational positioning scan (X-scan) with a peptide library where each epitope residue is consecutively replaced by every other amino acid (*85*, *86*). For each positive hit, peptide profiles are then searched in the human proteome to identify potential cross-reactive peptides. Unbiased methods have also been developed to assess TCR cross-reactivity. Positional scanning synthetic combinatorial libraries (PS-SCLs) consist of trillions of peptides where each natural amino acid is consecutively fixed at each position and the rest of the peptide is composed of a random combination of all other existing amino acids (*87*–*89*). Results from PS-SCL screening are then compared to protein databases to predict potential cross-reactive epitopes.

Recent studies performed high-throughput screening using peptide libraries and barcoded tetramers to test the recognition of thousands of peptides from the human proteome by selected TCRs (*51*, *90*). Although powerful, these screenings can only assess cross-reactivity for a subset of epitopes from the whole proteome. Other strategies based on three-dimensional (3D) modeling of pMHC-TCR complexes and structure-guided analysis were developed, alone or in combination with previous approaches to predict potential cross-reactivity considering the whole proteome as accurately as possible (*91*). Structure-guided design has been used to optimize TCR affinity with limited impact on specificity using x-ray crystal structure datasets to train prediction algorithms (*92*–*94*). Conversely, Hellman *et al.* (*95*) used structure-guided design to improve TCR specificity (limiting cross-reactivity) without altering TCR affinity. Other groups collected a dataset of known cross-reactivities and crystallographic structures to refine a 3D structural model, allowing prediction of cross-reactivity based on the similarity of electrostatic surfaces at the TCR-pMHC interface (*96*, *97*). Another dataset of TCR-pMHC interfaces was harnessed to develop and train an algorithm that demonstrated that TCR cross-reactivity with peptides without similar physicochemical properties was linked to structural flexibility of the pMHC complex (*98*, *99*). More recently, a deep learning–based prediction algorithm succeeded in predicting cross-reactivity with high sensitivity (*100*). Combining 3D modeling tools and peptide libraries associated with barcoded dextramers, Bentzen *et al.* (*51*) identified TCR fingerprints and epitope motifs underlying TCR-pMHC interaction. They observed that different TCRs targeting the same epitope mostly have different fingerprints, suggesting that each TCR-pMHC interaction should be considered unique. This concept was challenged by studies seeking to identify similarities within TCR targeting the same epitope (TCR clustering), where complementarity-determining region (CDR) similarities were used to regroup TCRs (*101*–*104*). As these studies aim at predicting antigens for a given TCR, they may provide useful information to assess cross-reactivity. Nonetheless, no clinical validation of these predictive approaches has been published. Implementation of the existing TCR-pMHC databases such as ATLAS (*105*) will be necessary to improve the prediction accuracy of these models. Additional and complementary approaches such as assessment of TCR-T cell cytotoxicity on primary cell lines (*86*) or assessment of the presence of pMHC complexes in situ in different tissues (immunopeptidomics and TCR-based histological analysis) may also help anticipate toxicities. A rationalized conception of TCR optimization must be implemented considering TCR affinity and avidity as well as efficiency and safety of TCR-T cells (*64*, *106*).

### Identification of resistance mechanisms

Primary and secondary resistance mechanisms to TCR-based immunotherapies have been described (*9*). Primary resistance mechanisms may be mainly represented by a low or a heterogenous expression of the target antigen in tumor cells or by intrinsic resistance of the tumor cells to T cell–mediated cytotoxicity (*107*). Another possible issue is the administration of most T cells with a late memory phenotype after the in vitro expansion step, leading to more exhaustion and less persistence than the infusion of T cell with a stem cell–like or an early memory phenotype (*108*). Regarding TCR-T cells, secondary or acquired resistance mechanisms represent the main concern. Up-regulation of immune checkpoint ligands at the surface of tumor cells can impair the expansion and functionality of the transferred T cells by activating immune checkpoint receptors, leading to exhaustion. The main escape mechanism to TCR-T cell therapies is the loss or decrease of MHC class I molecules on tumor cells, preventing the recognition of the target epitope by TCR-T cells. Loss of MHC class I expression can result from different mechanisms including deletion or mutations of HLA genes themselves, mutations of β*-2-microglobuline* or genes involved in antigen presentation, loss of heterozygosity, or epigenetic silencing of HLA genes in tumor cells. HLA-negative tumor cells would be positively selected during TCR-T treatment. Loss of HLA heterozygosity (HLA LOH) was recently described in two clinical trials using TILs specific for KRAS (*109*) or P53 (*110*) mutational neoepitopes. Another clinical trial targeting P53 mutations in a patient with breast cancer showed relapse 6 months after TCR-T cell therapy, with tumor cells expressing intact P53 and presenting LOH of chromosome 6 containing the HLA-A*02:01 locus (*34*). Epigenetic silencing of HLA genes was also described in a clinical trial where, after relapse, no mutation or LOH was detected by tumor sequencing, but treatment with the hypomethylating agent azacytidine restored the expression of HLA molecules (*111*). A recent clinical study using TCR-T cells targeting HPV-16 E7 antigen in HPV-16^+^ epithelial cancers showed that resistance to T cell therapies involved several actors of the antigen presentation process and of the interferon pathway, with a patient demonstrating loss of *TAP1*, *TAP2*, and *IFNGR*. Nonresponding or relapsed patients also showed impaired HLA-A*02:01 expression (*39*).

## PERSPECTIVES: NEXT STEPS IN T CELL ENGINEERING

As TCR-T cell therapies develop as a promising tool to target a wide panel of tumor antigens in solid tumors, many hurdles remain and need to be overcome, such as T cell–mediated toxicities, resistance mechanisms, and accessibility. To limit the risk of T cell activation against normal cells, it is theoretically possible to apply the strategies of logic gates proposed for CAR-T cells, where T cell activation or inhibition is conditioned by the integration of two signals instead of one. Logic gates “A AND B” are based on combinatorial antigen recognition and trigger the activation of the T cells only if both antigens A and B are concomitantly expressed by tumors cells. In “A NOT B” logic gates, normal cells that express an antigen B absent on tumor cells, in addition to the targeted antigen A, are protected (*112*). This approach is more challenging for TCRs compared to CARs, especially for the A AND B gates, but inhibitory signaling platforms have recently been developed (A NOT B gates) (*113*). Suicide gene systems aiming at creating a kill switch in engineered T cells as a safeguard mechanism are also an efficient strategy to stop unanticipated adverse events (*114*).

Besides toxicities, TCR-T cell therapies face many resistance mechanisms impairing their efficiency, as described above. Combination treatment of TCR-engineered T cells with ICIs can be achieved to avoid T cell exhaustion. In a clinical trial targeting P53 neoantigens (*34*), some patients were given pembrolizumab after the observation of a high fraction of PD-1^+^ antigen–specific T cells after infusion (*34*). Another clinical trial is testing a combination between MCPyV TCR-T cells and anti–PD-1/programmed cell death-ligand 1 treatments (*41*). Chapuis and colleagues (*115*) combined ACT of autologous antigen-specific T cells with cytotoxic T lymphocyte–associated antigen 4 blockade and demonstrated a durable clinical response, although the patient was refractory to both treatments individually. Last, gene modifications developed for CAR-T cell therapies to overcome tumor-related immunosuppression could also be used for TCR-T cells, such as PD-1 disruption, PD-1–CD28 chimeric constructs, or dominant negative transforming growth factor–β receptor type 2 (*116*). In addition to synthetic biology, combination with approaches targeting the tumor microenvironment (*7*, *8*) or modified cytokines resistant to hypoxic and acidic conditions prevalent in tumors may improve the efficiency of adoptive T cell therapies (*117*).

One of the main hurdles faced in T cell therapy clinical trials is the short persistence of adoptively transferred T cells related to the rapid exhaustion of engineered T cells (*115*). Generating engineered T cells with a stem cell memory phenotype (Tscm) at the time of infusion improves T cell persistence in vivo and long-term antitumor efficacy (*118*). It is possible to differentiate and expand Tscm in vitro starting from naive precursors (*119*). The supplementation of T cell culture medium with interleukin-7 (IL-7) and IL-15 enables the expansion of Tscm defined as CD45RA^+^ CD45RO^+^ CCR7^+^ CD62L^+^ CD95^+^ IL7RA^+^ (*108*). IL-21 can also support the Tscm phenotype of T cells during expansion (*120*). Although IL-2 is less efficient at maintaining a Tscm phenotype during the expansion phase, patient undergoing adoptive T cell therapy can be injected with IL-2 to support T cell proliferation and survival in vivo (*115*).

TCR-T cell therapies developed so far rely on engineering of autologous T cells. Similar to CAR-T cells, there would be many advantages in developing allogeneic approaches, such as immediate availability, possible standardization of the product, time for multiple cell modifications, easier redosing, and reduced cost (*116*). By deleting both endogenous TCRα and TCRβ chains, insertion of the transgenic TCR at the TRAC locus would avoid the risk of graft-versus-host disease. This technology should be combined with strategies to limit the rejection of the allogeneic T cells by the host immune system, such as partial HLA matching or gene editing (HLA class I deletion combined with natural killer cell inhibition) to generate universal T cells (*116*, *121*).

In conclusion, TCR-T cell therapy has already shown very encouraging results in solid tumors, including cancers responding poorly to current immunotherapies, such as sarcomas. The complexity of this therapeutic strategy is associated with many challenges. However, a better selection of TSAs and optimization of T cell engineering should reduce the risk of toxicity while increasing antitumor efficacy. Combination with therapeutics able to improve T cell homing (oncolytic viruses and radiation therapy) or increase the activity and persistence of the infused T cells (checkpoint inhibitors, cytokines, and cancer vaccines) may further increase the therapeutic potential of TCR-T cells. One can expect that this approach, like next-generation CAR-T cells, will change the natural history of cold tumors and provide a solution to a great therapeutic need.
